# A Microwave Three-Dimensional Imaging Method Based on Optimal Wave Spectrum Reconstruction

**DOI:** 10.3390/s20247306

**Published:** 2020-12-19

**Authors:** Yan Zhang, Baoping Wang, Yang Fang, Zuxun Song

**Affiliations:** 1 School of Electronic and Information, Northwestern Polytechnical University, Xi’an 710072, China; yan_jh@foxmail.com (Y.Z.); fangyang@mail.nwpu.edu.cn (Y.F.); zxsong@nwpu.edu.cn (Z.S.); 2 Science and Technology on UAV Laboratory, Northwestern Polytechnical University, Xi’an 710065, China

**Keywords:** microwave imaging, 3D imaging, sparse reconstruction, continuously distributed target, wave spectrum reconstruction

## Abstract

Limited by the Shannon–Nyquist sampling law, the number of antenna elements and echo signal data of the traditional microwave three-dimensional (3D) imaging system are extremely high. Compressed sensing imaging methods based on sparse representation of target scene can reduce the data sampling rate, but the dictionary matrix of these methods takes a lot of memory, and the imaging has poor quality for continuously distributed targets. For the above problems, a microwave 3D imaging method based on optimal wave spectrum reconstruction and optimization with target reflectance gradient is proposed in this paper. Based on the analysis of the spatial distribution characteristics of the target echo in the frequency domain, this method constructs an orthogonal projection reconstruction model for the wavefront to realize the optimal reconstruction of the target wave spectrum. Then, the inverse Fourier transform of the optimal target wave spectrum is optimized according to the law of the target reflectance gradient distribution. The proposed method has the advantages of less memory space and less computation time. What is more, the method has a better imaging quality for the continuously distributed target. The computer simulation experiment and microwave anechoic chamber measurement experiment verify the effectiveness of the proposed method.

## 1. Introduction

Microwave three-dimensional (3D) imaging is a kind of radar imaging technology that uses aperture synthesis to image the target in azimuth and altitude by transmitting and receiving the wide band electromagnetic wave signal. Different from traditional synthetic aperture radar imaging, microwave 3D imaging is generally used for high-resolution imaging of key targets. Because of its advantages of high-resolution, low-radiation, noncontact, and 3D imaging, it can be applied to security checks, medical diagnosis, disaster rescue, nondestructive testing, detection of hidden objects, and other fields [1,2,3,4,5,6,7,8,9,10,11,12,13].

Traditional time-domain integration 3D radar imaging methods, such as Back-Projection (BP), achieve focused 3D images by matching filtering, azimuth integration and altitude integration [14,15,16]. Traditional frequency-domain 3D radar imaging methods, such as range migration algorithm (or ω−k algorithm), which achieve 3D focused target images in a time-domain scene by correcting the radar echo space spectrum through frequency-domain interpolation. Traditional imaging methods have the advantages of stable performance and are widely used in practical imaging systems. The resolution of microwave imaging depends on the bandwidth, frequency, and synthetic aperture of the transmitted signal. Because of the limitation of the Shannon–Nyquist sampling law, the traditional imaging methods require the imaging system to have a very high sampling rate, which brings great difficulty to the hardware processing of the radar system. In addition, 3D imaging will produce a large amount of echo data, which also brings challenges to data processing [17,18,19,20,21].

Recently, many researches have introduced compressed sensing (CS) technology into microwave 3D imaging, which can greatly reduce the sampling rate and recover accurate target images from undersampled data [22,23,24]. The regularization CS imaging methods take the prior knowledge of the constraint, such as sparsity, which can further improve the imaging accuracy [25,26,27,28,29]. The total variation (TV)–regularized CS imaging method, which is sparsely represented by the energy difference between scattering points, can improve the antinoise performance of imaging, but reduces the imaging accuracy [30,31]. To improve both the imaging accuracy and the antinoise performance simultaneously, a hybrid constraint regularization CS imaging method based on joint TV-regularized and norm-regularized is proposed. This method adjusts the weight coefficients of TV constraint and norm constraint through an iterative cycle to achieve the optimal imaging results [32,33,34]. In addition, the results of these kinds of CS imaging methods are composed of a part of the discrete strong scattering points of the target, whereas the real target is often distributed continuously. Therefore, the imaging results are not good for the detailed features of the target, which is not conducive to the target recognition.

The spatial propagation characteristics and echo signal model of the target electromagnetic echo are analyzed in this paper. Based on the analysis of the effect of the target echo wave spectrum on imaging, the orthogonal projection model of target echo wavefront is constructed, and the optimal reconstruction of target echo wave spectrum is realized. Then, according to the gradient distribution of the target reflectance, the inverse Fourier transform (IFT) of the optimal target wave spectrum is iteratively optimized to improve the imaging accuracy. Computer simulation experiment and microwave anechoic experiment show that the method proposed in this paper can process sparsely sampled data; the memory consumption and calculation time are greatly reduced compared with the CS method, and the imaging accuracy is higher; especially the recovery of the detailed features of the target is more accurate.

## 2. Imaging Model

**Figure 1:**
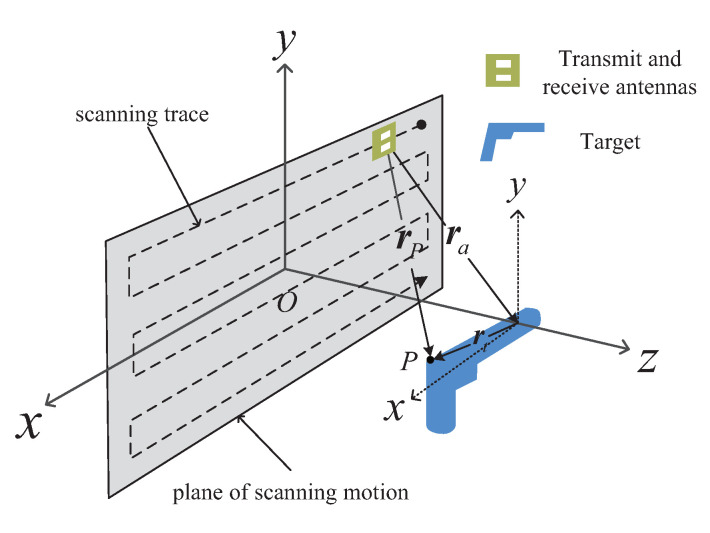
Imaging geometry.

In this paper, a planar scanning microwave 3D imaging system is discussed, and the Cartesian coordinate system is adopted to represent the geometric relationship of imaging, as shown in Figure 1. The radar scanning plane is xOy; the single input and single output model is adopted in radar working. Let $r_{t}\in R{\;}^{3}$ be the distance vector from the center of the target scene to a target point P, and $r_{a}\in \;R{\;}^{3}$ the distance vector from the radar antenna to the center of the target scene, and then the distance vector between the target point and the radar antenna is

(1) $$\begin{array}{c} r_{P}=r_{a}-r_{t} \end{array}$$

It is well known that if the imaging system is in free space and the medium of electromagnetic wave propagation is linear, isotropic, uniform, and nondispersive, then the electric field strength E and magnetic field strength H at $r_{P}$ satisfy the following Helmholtz equation:

(2) $$\begin{array}{c} \nabla^{2}E\left(r\right)\;+\;k^{2}E\left(r\right)=0 \end{array}$$

(3) $$\begin{array}{c} \nabla^{2}H\left(r\right)\;+\;k^{2}H\left(r\right)=0 \end{array}$$

where k=2ω/c is wave number, omega is the angular frequency of the transmitted signal, and *c* is the speed of light. In the Cartesian coordinate system, the orthogonal components of **E** and **H** satisfy the Helmholtz equation. Set the scattering point position of the target as $x_{t}$, $y_{t}$, $z_{t}$, and the scanning position coordinate of the antenna is $x_{a}$, $y_{a}$, $z_{a}$. Because the position coordinate $z_{a}$ of the antenna is a fixed value, and because the antenna transmits broadband waves containing multiple wave numbers at each scanning position, the variables of the echo level received by the radar are $x_{a}$, $y_{a}$, and *k*. Therefore, for the scattering point position of the target *P*, the general solution $E\left(x_{a},y_{a},k\right)$ can be represented by the following equation:

(4) $$\begin{array}{c} E\left(x_{a},y_{a},k\right)=\delta \left(P\right)e^{-jk2\left|r_{P}\right|} \end{array}$$

where $\delta \left(P\right)$ is the amplitude of the electric field intensity $E\left(x_{a},y_{a},k\right)$ of the echo of scattering point *P*. For 3D imaging, the target is equivalent to a set of multiple scattering points, so the radar echo is a set of all scattering point echoes in the target scene. Because the echo of different scattering points has the property of linear superposition, the echo signal of the whole target scene can be expressed as

(5) $$\begin{array}{c} E\left(x_{a},y_{a},k\right)=\;\int \int \int \;\delta \left(P\right)e^{-jk2\left|r_{P}\right|}dx_{t}dy_{t}dz_{t} \end{array}$$

## 3. Imaging Process

We would like to introduce our proposed method. In order to reduce the computational complexity and memory, the traditional three-dimensional imaging method based on inverse Fourier transform is used for reference. However, this method uses interpolation to complete the reconstruction of the spectrum, which will cause great errors and cannot process sparse sampled data. Our idea is to construct the dictionary matrix by orthogonal transformation and reconstruct the spatial spectrum satisfying the inverse Fourier transform, which can greatly improve the reconstruction accuracy and process the sparsely sampled data. For the problems of side lobe broadening and stray point interference in the imaging results, we optimize the imaging results by iterating the gradient target scattering information one by one, according to the gradient characteristics of the target reflectivity distribution.

### 3.1. Optimal Wave Spectral Reconstruction

What the radar sensor receives is the electromagnetic wave signal scattered from the irradiated target. If we regard the target as the signal source, the scanning plane of the radar antenna can be equivalent to a radar receiving plane $\overset{⌢}{r}$. Set $e_{x}$, $e_{y}$, $e_{z}$ as the unit direction vector parallel to the coordinate axis, and $x_{P},y_{P},z_{P}$ is the magnitude of the distance vector’s component on the coordinate axis, and then a distance vector between the target scattering point P and a certain antenna scanning can be expressed as

(6) $$\begin{array}{c} r_{n}=x_{n}e_{x}+y_{n}e_{y}+z_{n}e_{z}=\left(x_{a}-x_{t}\right)e_{x}+\left(y_{a}-y_{t}\right)e_{y}+\left(z_{a}-z_{t}\right)e_{z} \end{array}$$

For the sake of analysis, assume that the radar is emitting a single frequency wave. If the phase constant is $k_{l}$ (also known as wave number), then the electromagnetic wavefront at the radar receiving plane $\overset{⌢}{r}$ is

(7) $$\begin{array}{c} \Sigma \left(n\right)=e^{-jk_{l}r_{n}} \end{array}$$

By integrating the radar echo data of the scattering point P on the electromagnetic wavefront Σ, the reflectivity of the scattering point *P* of the target can be calculated as follows:

(8) $$\begin{array}{c} \delta \left(P\right)=\oint_{\Sigma \left(n\right)}Ed\Sigma \left(n\right) \end{array}$$

Under ideal conditions, the electromagnetic wave fronts (also known as equiphase planes) that propagate in any direction in the unbounded space of homogeneous media are perpendicular to the propagation direction. Therefore, at radar receiving plane $\overset{⌢}{r}$, the wavefront $\Sigma \left(n\right)$ of all electromagnetic waves is perpendicular to the distance vector $r_{n}$. Then, the front $\Sigma \left(n\right)$ of all the scattered electromagnetic waves passing through the radar receiving plane $\overset{⌢}{r}$ is a concentric surface with the target point as the center of the circle. Surface integration is very complicated in computer processing, so it is necessary to project the surface onto the plane. However, because the position coordinates of the target point are unknown, in order to facilitate the projection, it is necessary to represent $\overset{⌢}{\Sigma }$ in the coordinate $k_{x}-k_{y}-k_{z}$. Define $k_{l}$ as the wave vector in the same direction as $r\left(n\right);k_{x},k_{y},k_{z}$ is the component of $k_{l}$ in the direction $e_{x},e_{y},e_{z}$, as follows:

(9) $$\begin{array}{c} k_{l}=k_{x}+k_{y}+k_{z}=k_{x}e_{x}+k_{y}e_{y}+k_{z}e_{z} \end{array}$$

Then, the set $\overset{⌢}{\Sigma }$ of all wave vectors and wavefronts can be represented as follows:

(10) $$\begin{array}{c} \overset{⌢}{\Sigma }=e^{-jk_{l}r\left(n\right)}=e^{-jk_{l}\left(\left(x_{a}-x_{t}\right)e_{x}+\left(y_{a}-y_{t}\right)e_{y}+\left(z_{a}-z_{t}\right)e_{z}\right)}=e^{-j\left(\left(x_{a}-x_{t}\right)k_{x}+\left(y_{a}-y_{t}\right)k_{y}+\left(z_{a}-z_{t}\right)k_{z}\right)} \end{array}$$

In (10), $k_{l}$ is a fixed value. $k_{x}$ and $k_{y}$ can be determined according to the boundary conditions of the scanning plane, and $k_{z}$ is unknown. According to (10), $k_{z}$ is expressed in terms of variables $k_{x}$ and $k_{y}$ in the Cartesian coordinate system, and then wavefront can be expressed as

(11) $$\begin{array}{c} \overset{⌢}{\Sigma }=e^{-j\left(\left(x_{a}-x_{t}\right)k_{x}+\left(y_{a}-y_{t}\right)k_{y}+\left(z_{a}-z_{t}\right)\left(k_{l}-k_{x}-k_{y}\right)\right)} \end{array}$$

**Figure 2:**
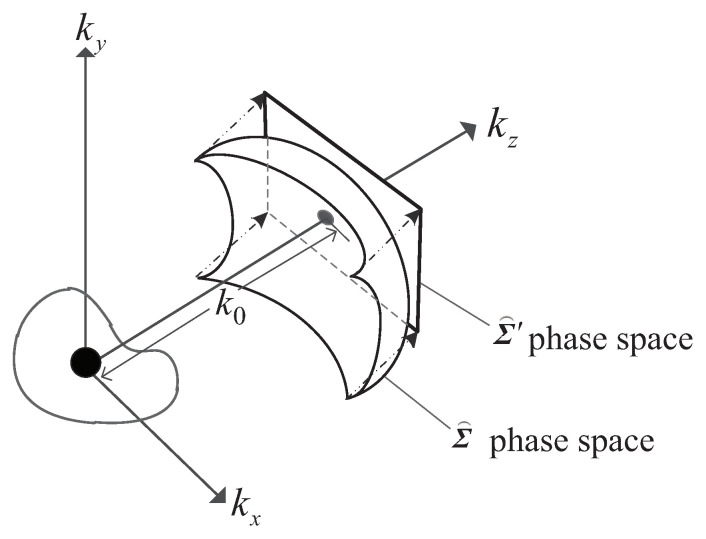
Schematic diagram of wavefront projection.

$\overset{⌢}{\Sigma }$ is still a surface in frame $k_{x}-k_{y}$, and the reflectivity of the target in the plane x−y cannot be obtained by IFT of the echo data on the $k_{x}$ axis and $k_{y}$ axis. Let $k_{0}=k_{l}e_{z}$ be the wave vector in the direction $e_{z}$; because $k_{l}$ is a fixed value, so $k_{0}$ is a constant vector, and its wavefront $\Sigma_{0}$ is a plane in the coordinate system $k_{x}-k_{y}$, as shown in Figure 2.

Let $k_{z}=k_{0}$, $\overset{⌢}{\Sigma }$ can be projected on the plane ${\overset{⌢}{\Sigma }}^{\prime }$, and then the new target echo wavefront is shown as follows:

(12) $$\begin{array}{c} {\overset{⌢}{\Sigma }}^{\prime }=e^{-j\left(\left(x_{a}-x_{t}\right)k_{x}+\left(y_{a}-y_{t}\right)k_{y}+\left(z_{a}-z_{t}\right)k_{0}\right)}=e^{-j\left(\left(x_{a}-x_{t}\right)k_{x}+\left(y_{a}-y_{t}\right)k_{y}+\left(z_{a}-z_{t}\right)k_{l}e_{z}\right)} \end{array}$$

Because $k_{l}$ is known, the radar echo data can realize projection transformation in the $k_{x}-k_{y}-k$ domain, so that the surface integral on ${\overset{⌢}{\Sigma }}^{\prime }$ can be transformed into the double integral of $k_{x}$ and $k_{y}$. At this point, the IFT is performed on the axis $k_{x}$ and axis $k_{y}$ to obtain the reflectivity of the target on the plane x−y. To obtain the reflectance distribution of the target on the axis *z*, the radar generally emits a broadband signal. Let *k* represent the wave number of broadband signals. Because the integral variables and data variables are in the same direction and are uncoupled, the echo data can be represented as a scalar in the Cartesian coordinate system, as follows:

(13) $$\begin{array}{c} F^{\prime }\left(k_{x},k_{y},k\right)=\;\int \int \int \;I\left(k_{x},k_{y},k\right)e^{-j\left(\left(x_{a}-x_{t}\right)k_{x}+\left(y_{a}-y_{t}\right)k_{y}+\left(z_{a}-z_{t}\right)k\right)}dk_{x}dk_{y}dk \end{array}$$

where $I\left(k_{x},k_{y},k\right)$ represents the spectral amplitude of the radar received data in the coordinate $k_{x}-k_{y}-k$. Next, the specific processing process is discussed according to the projection model, and the signal is represented as a matrix: s is defined as the original echo data matrix, $F=F\left(k_{x},k_{y},k\right)=F\left(s\right)$ is the wave spectrum matrix obtained by s through Fourier transform, and ${F_{i}}^{\prime }=F^{\prime }\left(k_{x},k_{y},k_{i}\right)$ is the wave spectrum matrix of the *i*th wavefront after projection transformation. Let $\Psi \left(\cdot \right)$ represent the transformation of the projection to F, and the relationship is as follows:

(14) $$\begin{array}{c} {F^{\prime }}_{i}=\Psi \left(F_{i}\right) \end{array}$$

Interpolation is one of the most used methods to realize projection transformation in computers, such as SINC interpolation, an interpolation method base on the SINC kernel function. However, interpolation has some errors and approximations. If the interpolation error is large, the image quality will be seriously reduced. In addition, if the radar imaging system adopts the method of random sparse sampling to collect data in the scanning plane and the transmitted signals, there are two problems. The first problem is that the data do not satisfy the Shannon–Nyquist sampling law. The second problem is that the data are not uniformly sampled. These two problems will cause the original echo data to be unable to use Fourier transform in the x direction and y direction, and the interpolation accuracy will be poor.

**Figure 3:**
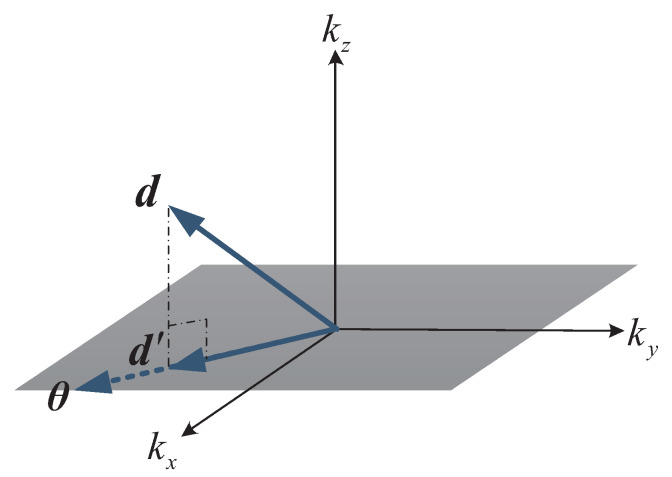
Schematic diagram of wavefront projection.

To calculate the optimal solution $F^{\prime }$ and to deal with the possible sparse sampling of emission waves, we build a sparse optimization model. We reconstruct the coordinate system based on the coincidence position of the original wave spectrum and the plane as the origin, as shown in Figure 3. Let d be a vector in the original wave spectrum $F_{i}$, θ is a vector on the plane $k=k_{z}$, and $d^{\prime }$ is the vector vertically projected by d on θ, then the relationship is as follows:

(15) $$\begin{array}{c} d^{\prime }=\alpha \theta \end{array}$$

where α is the projection coefficient of d onto θ. If $d^{\prime }$ is the vertical projection of d onto the plane $k=k_{z}$, then θ is the vector with the largest modulus multiplied by d on that plane. Then, according to the orthogonal projection relationship, the projection coefficient α can be expressed as follows:

(16) $$\begin{array}{c} \alpha =\frac{\theta^{T}d}{\theta^{T}\theta } \end{array}$$

If we construct a base space $G=\;\left[\begin{array}{cccc} g_{1} & g_{2} & \cdots & g_{L} \end{array}\right]$, so that $F_{i}$ and ${F^{\prime }}_{i}$ satisfy the orthogonal projection relation in Figure 3 on the base space $G_{i}$, ${F^{\prime }}_{i}$ can be solved by the method of orthogonal matching pursuit. In order to realize data transformation, the orthogonal projection basis matrix is constructed with a Gaussian function as a kernel function. Let q be the L-dimensional oversampling position vector, and the modulus of q is satisfied as follows:

(17) $$\begin{array}{c} k\;\leq \;\left|q\right|\;\leq \;{\left(k+k_{x}+k_{y}\right)}_{\max} \end{array}$$

If the position matrix is O = $q^{T}q$− Λ**q** in the way of oversampling, the vector g in the basis space can be represented as follows:

(18) $$\begin{array}{c} g={\left[\begin{array}{cccc} g_{1} & g_{2} & \cdots & g_{U} \end{array}\right]}^{T} \end{array}$$

where $g=e^{-o^{2}/b}$. Then, the relationship between $F_{i}$ and ${F^{\prime }}_{i}$ can be expressed as

(19) $$\begin{array}{c} F_{i}=G{F^{\prime }}_{i} \end{array}$$

If the Fourier basis of wave spectrum $F_{i}$ is $f_{}^{-}$, the relationship between $F_{i}$ and the original echo s can be expressed as

(20) $$\begin{array}{c} s=\Phi {F^{\prime }}_{i} \end{array}$$

where $\Theta =f^{-}G$. In order to facilitate matrix operation, $F_{i}$ is stretched into a one-dimensional matrix H, and set $H^{\prime }$ is the one-dimensional matrix after projection transformation, χ${}_{\lambda }$ is the residual matrix of iteration, Th is the residual threshold, and λ is the number of iterations. The specific solving process is shown in Table 1.

All the wavefront data of the echo signal are reconstructed iteratively through the Algorithm presented in Table 1, and the spectrum of the changed projection can be obtained. The reflectance distribution of the target can be obtained by pairing 3D-IFT.

### 3.2. Imaging Optimization Based on Reflectance Gradient Distribution

Through the optimal reconstruction of the target wave spectrum, the reflectance distribution (i.e., the target image) can be obtained through 3D-IFT. Although the imaging problem of sparsely sampled data can be solved, IFT will produce the main lobe broadening and the side lobe effect, resulting in the decline of the imaging quality. In order to take into account the continuous distribution characteristics and imaging accuracy of the target imaging results, we optimized the process of 3D-IFT by analyzing the sparse representation model of the target scene. The ideal wave spectral reconstruction imaging result of the isolated point target should be a 3D SINC function, but for the continuous distributed target, the reflectance distribution of the imaging result is related to the target material, geometric shape, noise interference, and so on. To analyze the variation characteristics of the target reflectance distribution, the 3D Hessian matrix is calculated for the target point, as follows:

(21) $$\begin{array}{c} Hessian\left[\delta \left(x,y,z\right)\right]=\left[\begin{array}{ccc} \frac{\partial \delta^{2}}{\partial x^{2}} & \frac{\partial \delta^{2}}{\partial x\partial y} & \frac{\partial \delta^{2}}{\partial x\partial z}\\ \frac{\partial \delta^{2}}{\partial y^{2}} & \frac{\partial \delta^{2}}{\partial y\partial x} & \frac{\partial \delta^{2}}{\partial y\partial z}\\ \frac{\partial \delta^{2}}{\partial z^{2}} & \frac{\partial \delta^{2}}{\partial z\partial x} & \frac{\partial \delta^{2}}{\partial z\partial y} \end{array}\right] \end{array}$$

**Figure 4:**
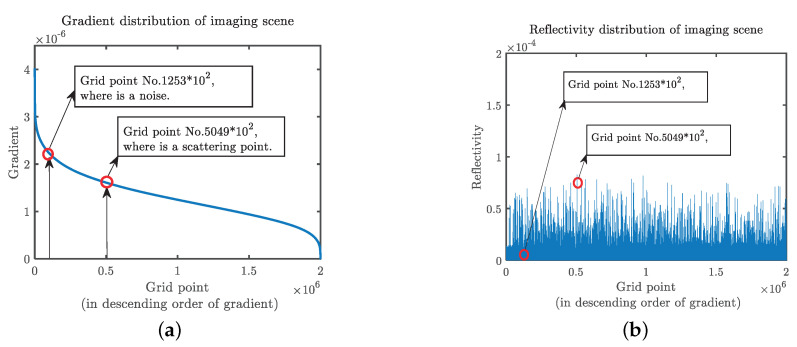
Diagram of target scene reflectance distribution. (**a**) Gradient distribution of imaging scene. (**b**) Reflectivity distribution of imaging scene.

$Hessian\left[\delta \left(x,y,z\right)\right]$ represents the gradient of target reflectance in different directions in 3D space, which is the intensity of $\delta \left(x,y,z\right)$ change. In the target RCS measurement, continuous distribution gradient distribution generally has the following features: when $\delta \left(x,y,z\right)$’s gradient values in all directions are bigger, it means that point $\left(x,y,z\right)$ is the strong scattering point or strong stray points, whereas the strong scattering and stray points have similar gradient distribution characteristics, but the strong scattering point generally has a higher reflectivity. The distribution of reflectivity amplitude and gradient of the imaging result of a target containing noise is represented by the imaging scene grid points, and the imaging scene is stretched into a one-dimensional matrix, as shown in Figure 4. When the gradient value of $\delta \left(x,y,z\right)$ is large in only a few directions, it means that the point $\left(x,y,z\right)$ is located at the edge of the target material or geometric structure. When the gradient value of O is small in all directions, it means that the point $\left(x,y,z\right)$ is located in the region where the material or geometric structure of the target is uniformly distributed.

To reduce the interference of the stray points to the imaging results, the reflectance distribution and the gradient distribution of the target can be analyzed simultaneously. For the convenience of calculation, 1 norm ${∥Hessian\left[\delta \left(x,y,z\right)\right]∥}_{1}$ of the Hessian matrix is used to characterize the global gradient value of the target point. By setting certain threshold values $T_{\delta }$ and $T_{H}$, the scattering points with a low $\delta \left(x,y,z\right)$ but high ${∥Hessian\left[\delta \left(x,y,z\right)\right]∥}_{1}$ can be filtered out. In the postprocessing of traditional radar imaging results, the image is generally windowed with a drop of 3 dB as the threshold. Although this can improve the peak signal-to-noise ratio (PSNR) of the image to some extent, it will also annihilate the weak scattering points. According to the characteristics of the reflectance distribution and gradient distribution of the target, cyclic optimization was carried out in an iterative manner. In each optimization, the strongest scattering point was found, and the boundary of the target reflectance distribution was found by calculating the Hessian matrix. The image inside the boundary was extracted and filtered in the original spectral distribution until the optimal imaging result was calculated. If the sparsity κ of the target scene is known, it can be used as the stop iteration threshold. In the actual imaging scenario, κ needs to be estimated. One of the estimation methods is to analyze the approximate image, and the approximate image of the target scene can be obtained by sparse projection. The specific process is as follows:

(1) The reflectivity $\delta \left(x,y,z\right)$ of the target imaging results and the 1 norm ${∥Hessian\left[\delta \left(x,y,z\right)\right]∥}_{1}$ of the Hessian matrix are sorted, respectively. If $\delta \left(x,y,z\right)<T_{\delta }$ and ${∥Hessian\left[\delta \left(x,y,z\right)\right]∥}_{1}>T_{H}$, the reflectivity value of the point is set to zero.

(2) Start the iteration, initialize the number of iterations λ=0, find the point with the highest reflectance, and then find the main lobe boundary by calculating the extreme value point of the Hessian matrix, and save the reflectance distribution $\delta^{\prime }\left(x,y,z\right)$ of all target points within the boundary in the final imaging result $\hat{I}$;

(3) Make 3D-FT for $\delta^{\prime }\left(x,y,z\right)$, and delete the result from the original spectral distribution $F^{\prime }$;

(4) Let iteration times be λ=λ+1. If λ<κ, return to Step 2;

otherwise, stop the iteration, and get the final image result $\hat{I}$.

Compared with the traditional imaging method, which windows the imaging results directly, the iterative optimization method proposed in this paper not only can improve the PSNR of the image, but also can retain the information of weak scattering points better. In addition, the method uses a Hessian matrix to calculate the boundary of reflectance gradient distribution of scattering points, so as to retain the main lobe information of scattering points, so as not to destroy the characteristics of continuous distribution of targets in the imaging results.

## 4. Experimental Results

We conduct three experiments to evaluate the efficacy of our proposed method. Firstly, we use simulation data to evaluate the efficacy of the two steps in the proposed method. Then, we use the echo data of five metal sphere targets collected in the microwave chamber to analyze the imaging effect, calculation time and memory occupation of the proposed method. Finally, we analyze the imaging effect of the proposed method on continuous distribution targets by the combined target collected in a microwave chamber.

### 4.1. Experimental Analysis with Simulation Data

**Figure 5:**
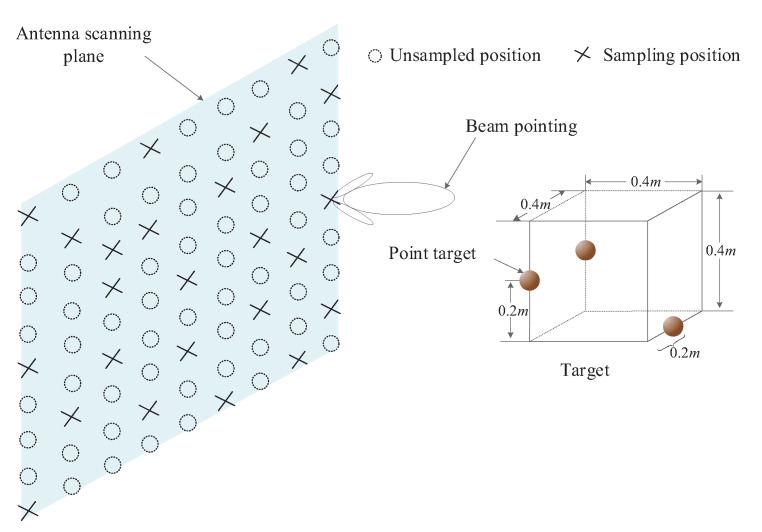
Computer simulation of the target model.

Based on the mathematical model of (5), this section analyzes and verifies the key steps of the method proposed in this paper through computer-generated simulation experimental data. Although there is a big difference between the computer simulation data and the real echo data, compared with the real echo data, the computer simulation data are easier to adjust and control the experimental conditions and have completely known experimental parameters. In the computer simulation experiment, the target is composed of three scattering points. The radar scanning plane is 5 m away from the target scene center, the radar transmitting frequency is 12 to 16 GHz, the frequency stepping is 0.02 GHz, and the horizontal and vertical scanning distance is 2 m. The target model is shown in Figure 5, and the direction of radar wave emission is shown in Figure 5, and the direction of radar wave emission is x.

**Figure 6:**
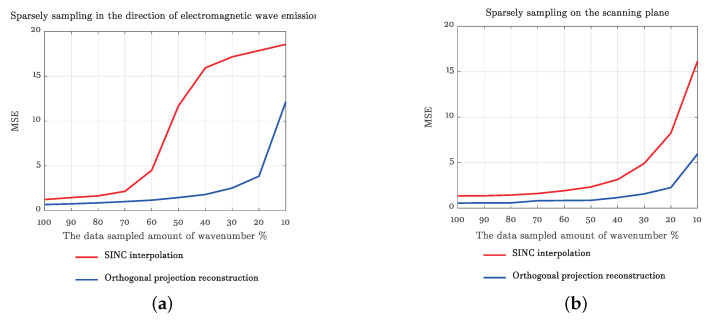
Wave spectrum transformation mean square error curves. (**a**) Sparsely sampling in the direction of electromagnetic wave emission. (**b**) Sparsely sampling on the scanning plane.

The accuracy of the target wave spectrum transformation is crucial to the imaging quality. The echo signals are sparsely sampled in the frequency (a correspondence with wave number) and the scanning plane, respectively, and the mean square error (MSE) at different sparse sampling rates is shown in Figure 6. As can be seen from Figure 6a, for radar transmitting direction, the mean square error of SINC interpolation increases slowly when the sparse sampling rate is larger than 30%, which is slightly larger than the method proposed in this paper. The mean square error increases rapidly when the sparse sampling rate is less than 30%. In addition, the mean square error of the method proposed in this article is significantly smaller than that of SINC interpolation when the sparse sampling rate is large and 20%. As can be seen from Figure 6b, for the scanning plane, the mean square error of SINC interpolation increases slightly when the sparse sampling rate is less than 70% and increases rapidly when the sparse sampling rate is less than 70%. The mean square error of the method proposed in this paper increases slowly when the sampling rate is large and 30% and increases rapidly when the sparse sampling rate is less than 30%. By comparing Figure 6a,b, for SINC interpolation, the influence of sparse sampling of echo data in the direction of radar transmission is significantly less than that of sparse sampling in the scanning plane.

**Figure 7:**
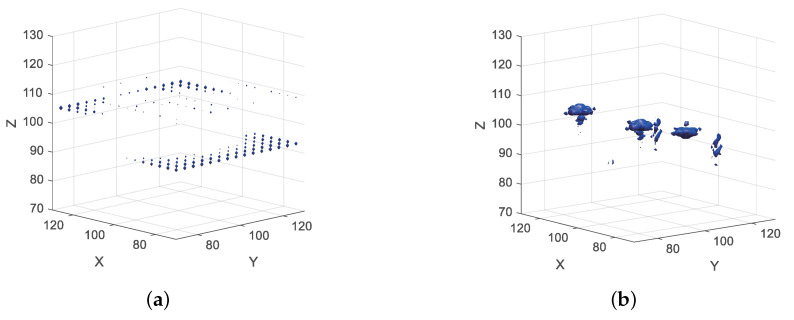
Three-dimensional inverse fast Fourier transform (IFFT) imaging results of 30% scan plane sparse sampling data. (**a**) SINC interpolation. (**b**) Orthogonal projection reconstruction.

In order to intuitively verify the impact of different spectral reconstruction methods on imaging under the condition of sparse sampling on the scanning plane, we used the echo data of sparse sampling on the 30% scanning plane for verification. Figure 7 shows the imaging results of inverse fast Fourier transform (IFFT) directly after wave spectrum transformation. It can be seen from Figure 7a that the imaging results of wave spectrum transformation realized by SINC interpolation were completely defocused. As shown in Figure 7b, although there were some defocus and high side lobe in the imaging results, in which the wave spectrum transformation is realized by orthogonal projection reconstruction, the three scattering points could be well distinguished. It means that the orthogonal projection reconstruction method proposed in this paper still has a good reconstruction accuracy under 30% scan plane sparse sampling and can meet the imaging requirements.

**Figure 8:**
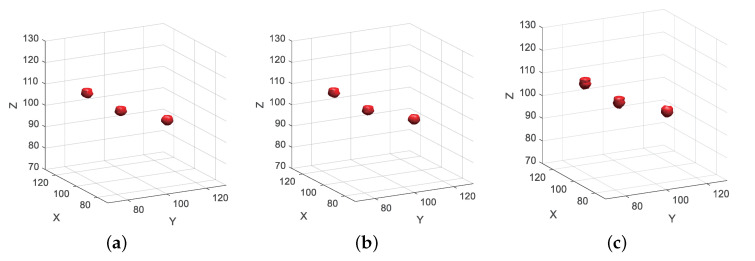
Imaging results with different sample rate data. (**a**) 100% sampled data. (**b**) 25% wave number sampling data. (**c**) 25% scan plane sampling data.

Figure 8 is the imaging result with a complete process of the proposed method. Figure 8a is the imaging result that used 100% sampling data. Compared with the target model of Figure 6, the imaging results achieved an accurate reconstruction of the target reflectivity distribution, because of the imaging based on reflectivity gradient distribution optimization, so the scattering point imaging results of the side lobe gain better inhibition. Figure 8b is the imaging result of sparsely sampled data of 30% transmitted wave number, which is basically unchanged compared with Figure 8a. Figure 8c is the imaging result of sparsely sampled data of 30% scan plane. Compared with Figure 8a,b, the space volume of reconstruction result of each scattering point is larger. However, compared with Figure 7b, the side lobe is well filtered and suppressed, and the positions of the three scattering points can be clearly distinguished, indicating that the proposed method significantly improves the imaging quality of the data with low scan plane sparse sampling rate.

### 4.2. Experiment of Measured Data in Anechoic Chamber

**Figure 9:**
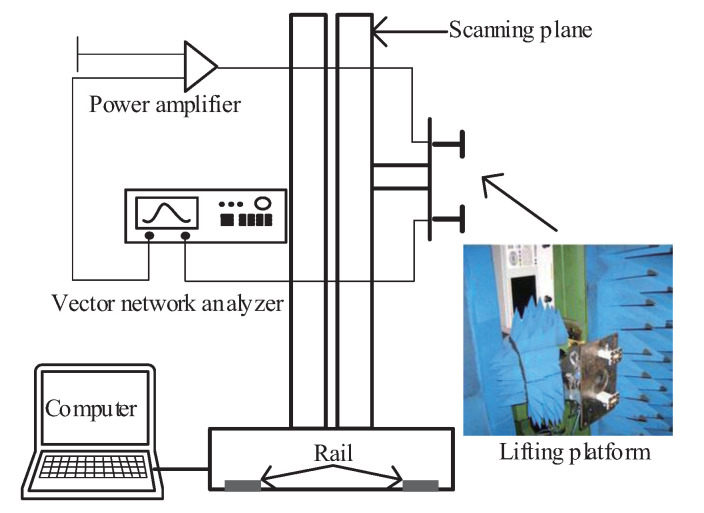
Anechoic chamber test scheme.

**Figure 10:**
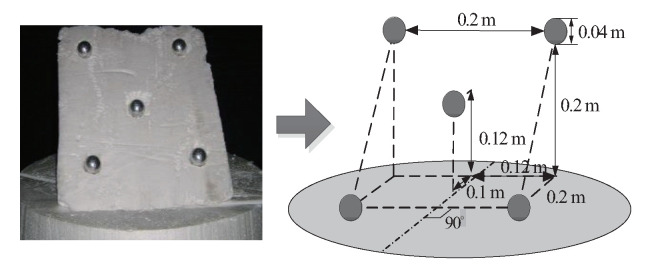
Metallic balls target model.

In order to test the effectiveness of the proposed method in the real radar imaging experiment, this study built a microwave anechoic chamber semitest platform for a planar scanning 3D imaging test experiment. The test system is shown in Figure 9. A radar system was simulated by means of a vector network analyzer, a power amplifier, a transmitting probe, and a receiving probe. The simulated radar system was placed on a lifting scanning frame, and the scanning frame was placed on a guide rail. The plane scanning was realized by lifting the scanning frame and moving along the guide rail. The test was carried out by means of a series of scanning and a first-round collection. The frequency of the transmitted wave was 8 to 12 GHz, the frequency sampling interval was 0.04 GHz, the scanning interval was 0.02 m, and the vertical distance between the scanning plane and the target center was 2 m. A target composed of five metal balls is used for testing. The geometric position relation of the target is shown in Figure 10.

In order to analyze the advantages of the proposed method by indexes, the structural similarity (SSIM) and reconstruction error are used to evaluate the proposed method and other methods.

The SSIM [35] is commonly used for 2-D image quality assessment. It is considered more consistent with human eye perception than either the PSNR or the MSE. Hence, the specific form of the SSIM index between signals x and y is defined as

(22) $$\begin{array}{c} SSIM\left(x,y\right)=\frac{\left(2\mu_{x}\mu_{y}+C_{1}\right)\left(2\sigma_{xy}+C_{2}\right)}{\left(\mu_{x}^{2}+\mu_{x}^{2}+C_{1}\right)\left(\sigma_{x}^{2}+\sigma_{y}^{2}+C_{2}\right)} \end{array}$$

where $C_{1}$ and $C_{2}$ are the auxiliary variables, and $\mu_{x}$($\mu_{y}$), $\sigma_{x}^{2}$($\sigma_{y}^{2}$) and $\sigma_{xy}$ are the weighted mean, variance, and covariance, respectively, which are computed locally in a cubic window with a weighting function. The cubic window moves pixel-by-pixel over the entire 3-D image. Throughout these simulations, the SSIM measure uses the following typical parameter settings: $C_{1}=10^{-4}$, $C_{2}=9\times 10^{-4}$.

Reconstruction errors can describe the accuracy of reconstruction, which are characterized by reconstructed images I and original echo signals s, as shown in the following expression

(23) $$\begin{array}{c} Error=\frac{{∥s-\mathrm{DI}∥}_{1}}{{∥s∥}_{1}} \end{array}$$

where D is the dictionary matrix constructed by the echo phase, which refer to (5).

Table 2 shows the computer memory space, computation time occupied and SSIM by the frequency interpolation method, CS method, and proposed method under the condition of 100% sampling data. The echo data are 51 × 51 × 101. For CS imaging, the target scene discrete grid is 41 × 41 × 41. The hardware processing platform used in this experiment is a common desktop computer with an Intel Core i7-8700k processor. The software platform is MATLAB 2017. It can be seen from the table that the frequency-domain interpolation method has the lowest memory footprint and the lowest computing time. The OMP-CS method and $\ell_{1}$-TV-CS method takes up a huge amount of memory and computing time. The method proposed in this article takes up less memory and is basically in the same order of magnitude as the frequency-domain interpolation method. Although the calculation time is higher than the frequency-domain interpolation method, it is still far less than the two CS methods. The proposed method in this paper has the highest SSIM and lowest error, followed by the $\ell_{1}$-TV-CS method and frequency domain interpolation method, while the OMP reconstruction method has the lowest SSIM and highest SSIM. Through SSIM and error, it can be seen that the proposed method has a high accuracy in the geometric shape and reconstruction of the target.

**Figure 11:**
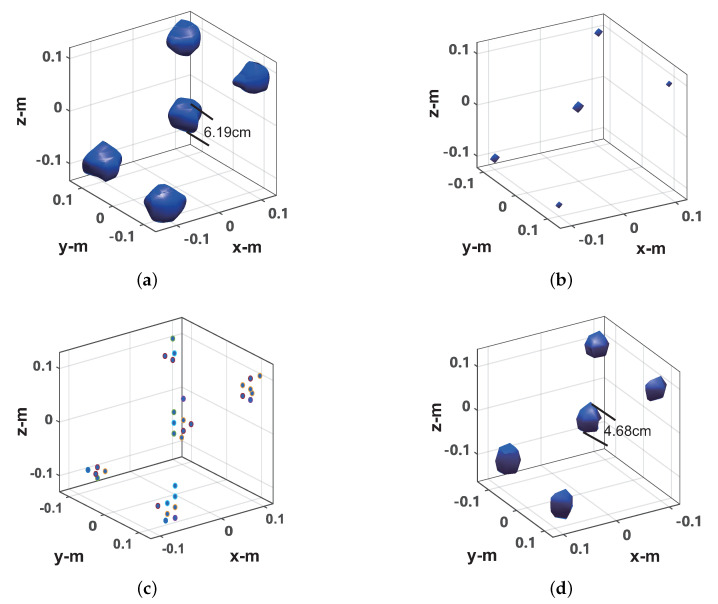
Imaging results from 100% sampled data. (**a**) Frequency interpolation method. (**b**) OMP-CS method. (**c**) $\ell_{1}$-TV-CS method. (**d**) Proposed method.

**Figure 12:**
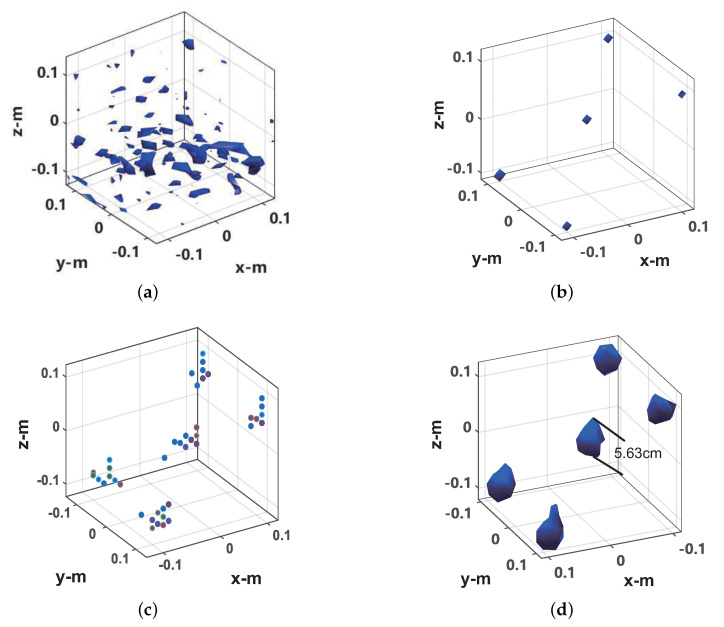
Imaging results from 30% sampled data. (**a**) Frequency interpolation method. (**b**) OMP-CS method. (**c**) $\ell_{1}$-TV-CS method. (**d**) Proposed method.

Figure 11 and Figure 12 show the imaging results of 100% sampling and 30% random sampling of the data on the scanning plane (used to simulate random sparse sampling, which was adopted in the microwave anechoic chamber data sparse imaging experiment), respectively. The image display threshold is −5 dB of the image peak value. For CS imaging, the imaging scene size was set to 0.4 × 0.4 × 0.4 m, and the imaging scene discrete grid was transformed into 21 × 21 × 21. As can be seen from Figure 11a, the diameter of the central metal sphere is approximately 6.19 mm in the imaging result of the frequency interpolation method. Compared with the actual metal sphere radius (4 mm), there is a relatively obvious expansion. As can be seen from Figure 11b, in the imaging results of the sparse CS imaging method based on the target scene, each ball is a discrete point. As can be seen from Figure 11c, in the imaging results of the $\ell_{1}$-TV-CS method, each metal ball consists of several scattered points. As can be seen from Figure 11d, in the imaging results of the proposed method, the diameter of the central metal ball is 4.68 mm, which is only slightly wider than the actual radius of the metal ball. As can be seen from Figure 12a, for 30% scan plane sparse data, the imaging result of the frequency interpolation method is completely defocused. As can be seen from Figure 12b, the imaging results of the OMP-CS imaging method still maintain a good quality. As can be seen from Figure 12c, in the imaging results of the $\ell_{1}$-TV-CS method, the focus of the target has decreased. It can be seen from Figure 12d that the proposed method can still perform better imaging of the target, and the imaging result has a small broadening compared with the imaging result of 100% sampling data.

**Figure 13:**
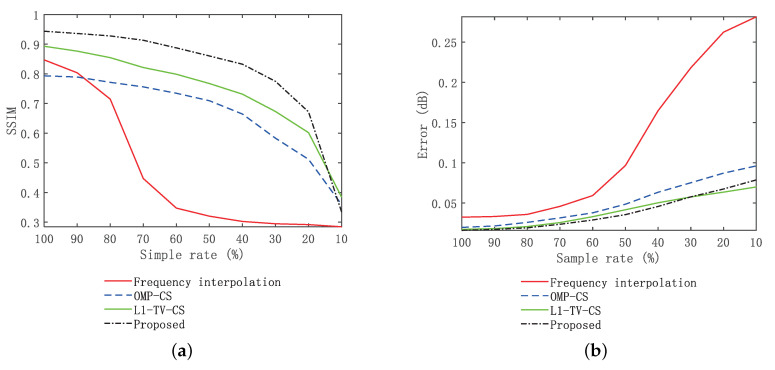
Imaging performance at different sample rates. (**a**) Frequency interpolation method. (**b**) SSIM curve analysis of imaging results.

Figure 13 shows the SSIM and error curves of the four methods with different sparsity. It can be seen from Figure 13a that under the condition of 100% sampling data, the proposed method has the highest SSIM value, whereas the CS method is slightly lower than the proposed method. The SSIM value for the frequency interpolation method is approximately 0.8. With the increase of sparsity, the OMP-CS method and $\ell_{1}$-TV-CS method keep the best stability. The SSIM value of the frequency interpolation method drops significantly. However, the method in this paper can maintain good stability when the sparsity is more than 30%, and the decrease is more obvious when the sparsity is less than 30%. The combination of Figure 13 indicates that the proposed method is slightly more sensitive to random sparse sampling of data than the OMP-CS method and $\ell_{1}$-TV-CS method, but significantly lower than the frequency interpolation method. It can be seen from Figure 13a that the reconstruction accuracy of the frequency interpolation method is poor in low sampling rate conditions, the proposed method has obvious advantages over the OMP-CS method and $\ell_{1}$-TV-CS method at a sampling rate of 100–30%.

**Figure 14:**
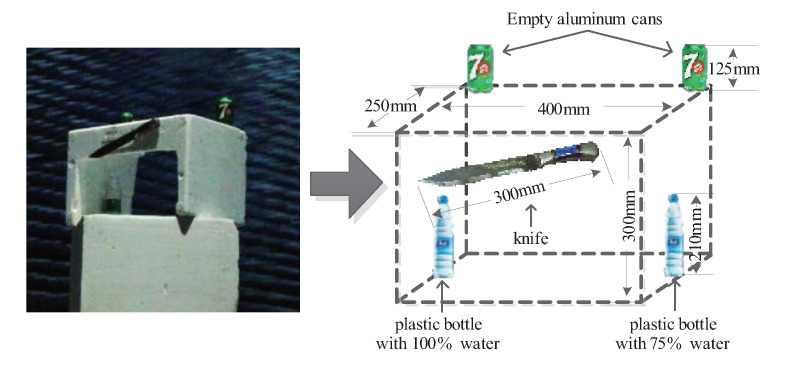
Assembly target model.

To further verify the imaging effect of the proposed method on continuously distributed targets, a knife, two mineral water bottles, and two cans are selected in this section to form a target body, as shown in Figure 14. The objects were taped to a Styrofoam holder with a metal handle and blade. The size of the two water bottles is the same, but the volume of the water is different; the left side of the mineral water filled with water to the mouth of the bottle, and the right side of approximately three-fourths full of water. The two cans contained nothing. The radar simulation test system still adopts the test scheme shown in Figure 9.

**Figure 15:**
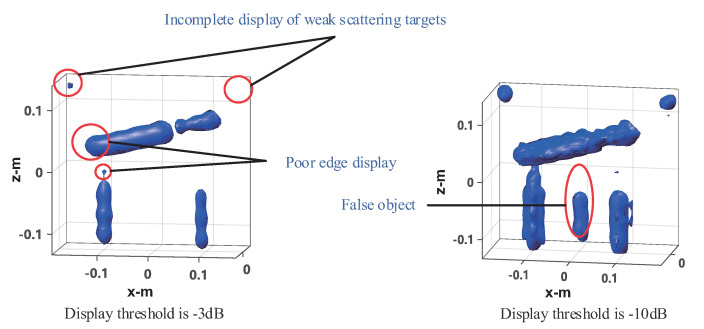
Imaging results of the frequency interpolation method with different display threshold.

**Figure 16:**
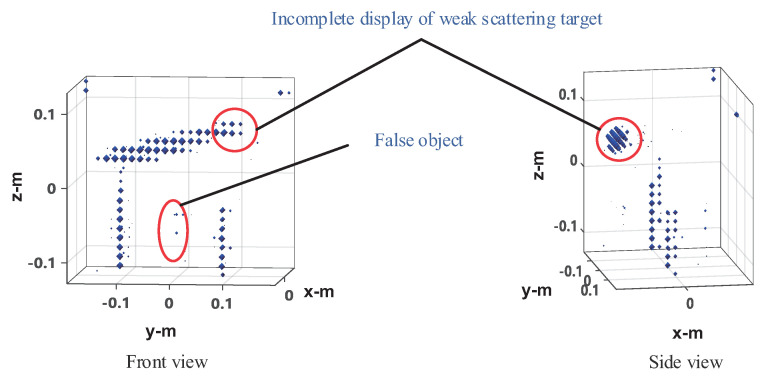
Imaging results of the OMP-CS method.

**Figure 17:**
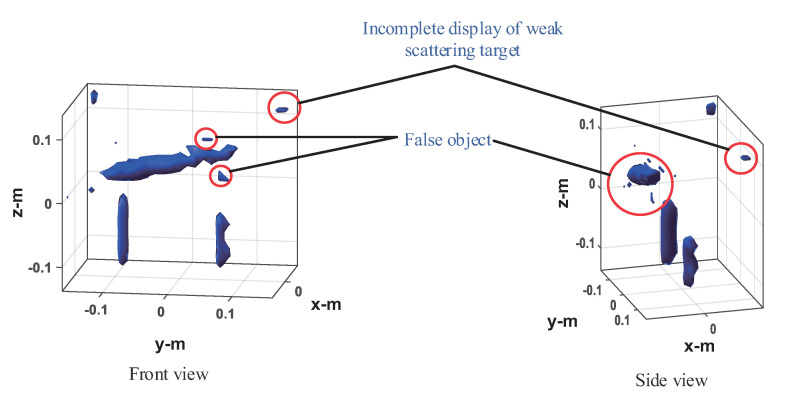
Imaging results of the $\ell_{1}$-TV-CS method.

**Figure 18:**
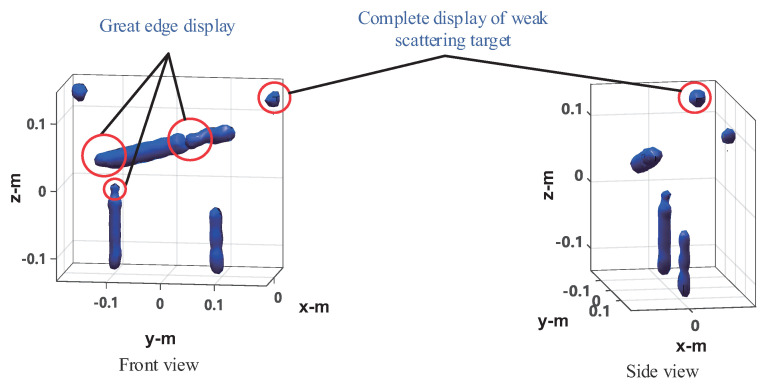
Imaging results of the proposed method.

The four method’s performances are verified under 50% sampling data. The imaging results of the frequency interpolation method are shown in Figure 15. Left is the imaging result with the peak image energy decreasing by 3 dB as the display threshold. As can be seen from the figure, the imaging results of the knife and the mineral water bottle are close to the real target, but there is some distortion, especially the tip of the blade and the opening of the bottle of the mineral water on the left, as shown in the black circle. The height of the water bottle on the right is slightly lower than that on the left. This is because the reflection ability of the electromagnetic wave of plastic is very weak, whereas the reflection ability of the electromagnetic wave of water is stronger. The imaging result is actually the water in the bottle. The can on the left is visible, but it shows a tiny dot, whereas the can on the right shows nothing at all. This indicates that the electromagnetic wave reflection ability of empty cans is weak, and most of the reflected energy is lower than the −3 dB threshold. On the right is the imaging result with the image energy peak decreasing by 10 dB as the display threshold. As can be seen from the figure, both the cans can be fully displayed, but at the same time, the side lobe of the strongly scattered object (knife and water in the mineral water bottle) is higher, and there is a false target between the two mineral water bottles, resulting in a serious decline in the image quality. In the CS imaging processing, the size of the imaging scene is 0.6 × 0.4 × 0.4 m, and the discrete mesh of the imaging scene is transformed into 31 × 21 × 21. Here we use OMP and $\ell_{1}$-TV reconstruction algorithms, respectively, for imaging experiments. The imaging results of OMP reconstruction are shown in Figure 16. Knives, mineral water bottles, and cans can all be displayed with less stray points, and no side lobe interference to the imaging results. However, as the imaging results are composed of discrete points, they can only reflect the general outline of the target, and the details of the target shape are poorly reflected, which is not conducive to target recognition. The imaging results of $\ell_{1}$-TV reconstruction are shown in Figure 17. Compared to the OMP reconstruction results, $\ell_{1}$-TV reconstruction results have better continuity, but there are some noise and false objects in the image. Figure 18 is the imaging result of the proposed method. Compared with the frequency interpolation method, the imaging result of the proposed method is closer to the contour of the real target. The contour characteristics of the edge tip and the left edge of the mineral water bottle can be clearly seen, as shown in the black circle in the figure. The two cans are clearly visible in the image, and there are few stray points and side lobes to interfere with the imaging results.

## 5. Conclusions

This article proposes a microwave 3D imaging method gradient distribution optimization based on the optimal wave spectrum reconstruction and target reflectivity gradient distribution optimization. First, the orthogonal projection model is used to express the target wave spectrum, so as to achieve the optimal reconstruction of the target wave spectrum, and then the IFT of the target wave spectrum is iterated and optimized according to the distribution characteristics of the target reflectance gradient, so as to obtain high-quality imaging results. The proposed method is verified and analyzed by a computer simulation experiment and microwave anechoic chamber experiment. The visual perception and the comparison of SSIM and error parameter show that when the data sampling rate is 30–100%, the imaging quality of the proposed method is better than that of the frequency interpolation method, OMP-CS method and $\ell_{1}$-TV-CS method. In addition, the reduction of memory and calculation time is one of the great advantages of the proposed method, the memory of the proposed method is about 98% lower than that of the OMP-CS method and $\ell_{1}$-TV-CS method, the computation time of the proposed method is about 88% lower than that of the OMP-CS method and 97% lower than that of the $\ell_{1}$-TV-CS method.

## Figures and Tables

**Table 1:** Algorithm.

Algorithm	Optimal Wave Spectrum Reconstruction	
Input:		**H**, Θ
Initialize:		$\chi_{\lambda }=$H, $\Pi_{\rho }=0$, λ=0
Iteration:
	Step 1:	Calculate the maximum projection vector:
		$$\Theta_{\eta }=\max\left(\Theta_{\eta }H\right),\;\;\;\;\;\eta =1,2,3,\ldots$$
	Step 2;	Calculate projection coefficient:
		$$\alpha_{\eta }=\frac{\Theta_{\eta }^{T}H}{\Theta_{\eta }^{T}\Theta_{\eta }}$$
	Step 3:	Fill the results into optimal wave spectral matrix:
		$${H^{\prime }}_{\eta }=\alpha_{\eta }\Theta$$
	Step 4;	Calculation of residual:
		$$\chi_{\lambda }=F-\alpha_{\eta }\Theta_{\eta }$$
	Step 5:	If $\left|\chi \right|$ ≤Th, Stop the iteration;
		Otherwise, return to step 1.
Output:		**H’**

**Table 2:** Imaging performance comparison.

	Frequency Interpolation	OMP-CS	$\ell_{1}$-TV-CS	Proposed
Memory (Gb)	0.580	269.804	270.374	2.120
Computing time (s)	11.870	1802.241	9012.472	203.133
SSIM	0.849	0.792	0.913	0.947
Error (dB)	3.24 $\times \;10^{-2}$	1.97 $\times \;10^{-2}$	1.73 $\times \;10^{-2}$	0.165 $\times \;10^{-2}$
